# CHDGKB: a knowledgebase for systematic understanding of genetic variations associated with non-syndromic congenital heart disease

**DOI:** 10.1093/database/baaa048

**Published:** 2020-07-01

**Authors:** Lan Yang, Yang Yang, Xingyun Liu, Yongquan Chen, Yalan Chen, Yuxin Lin, Yan Sun, Bairong Shen

**Affiliations:** 1 Center for Systems Biology, Soochow University, Suzhou 215006, China; 2 Center of Prenatal Diagnosis, Wuxi Maternal and Child Health Hospital Affiliated to Nanjing Medical University, Wuxi 214002, China; 3 School of Computer Science and Technology, Soochow University, Suzhou 215006, China; 4 Institutes for Systems Genetics, West China Hospital, Sichuan University, Chengdu 610041, China

## Abstract

Congenital heart disease (CHD) is one of the most common birth defects, with complex genetic and environmental etiologies. The reports of genetic variation associated with CHD have increased dramatically in recent years due to the revolutionary development of molecular technology. However, CHD is a heterogeneous disease, and its genetic origins remain inconclusive in most patients. Here we present a database of genetic variations for non-syndromic CHD (NS-CHD). By manually literature extraction and analyses, 5345 NS-CHD-associated genetic variations were collected, curated and stored in the public online database. The objective of our database is to provide the most comprehensive updates on NS-CHD genetic research and to aid systematic analyses of pathogenesis of NS-CHD in molecular level and the correlation between NS-CHD genotypes and phenotypes.

**Database URL**: http://www.sysbio.org.cn/CHDGKB/

## Introduction

Congenital heart disease (CHD) is one of the most prevalent birth defects, ranging from 6.9 per 1000 births in Europe to 9.3 per 1000 births in Asia (1, 2). Although the CHD survival rate has improved due to the recent rapid development of surgical repair, not all cardiac defects can be diagnosed by a routine prenatal ultrasound. The mortality rate among patients with severe CHD remains high. While environmental factors have been shown to greatly contribute to the onset and progression of CHD (3), the huge impact of genetic defects on the pathogenesis of CHD during cardiac development has also been well documented (4–8). When exposed to the same environment, different individuals exhibited variable susceptibilities to CHD, suggesting the effect of hereditary factors. The main known genetic factors leading to CHD include focal mutation and chromosomal abnormalities (9). Nevertheless, the genetic origin of CHD, as well as the correlation between its genotypes and phenotypes, remains unclear. As studies in recent decades have mainly focused on the effect of cardiac surgery on CHD patients (10) or the incidence of CHD subtypes with genetic variations (11) at a systematic analysis level, there is no available database associated with genetic etiology of CHD. The database presented in our current study provides investigators and the public with a systematic and comprehensive understanding of the genetic etiologies of non-syndromic CHD (NS-CHD) to improve the interpretation of the NS-CHD variants.

## Methods

### Data collection

**Figure 1:**
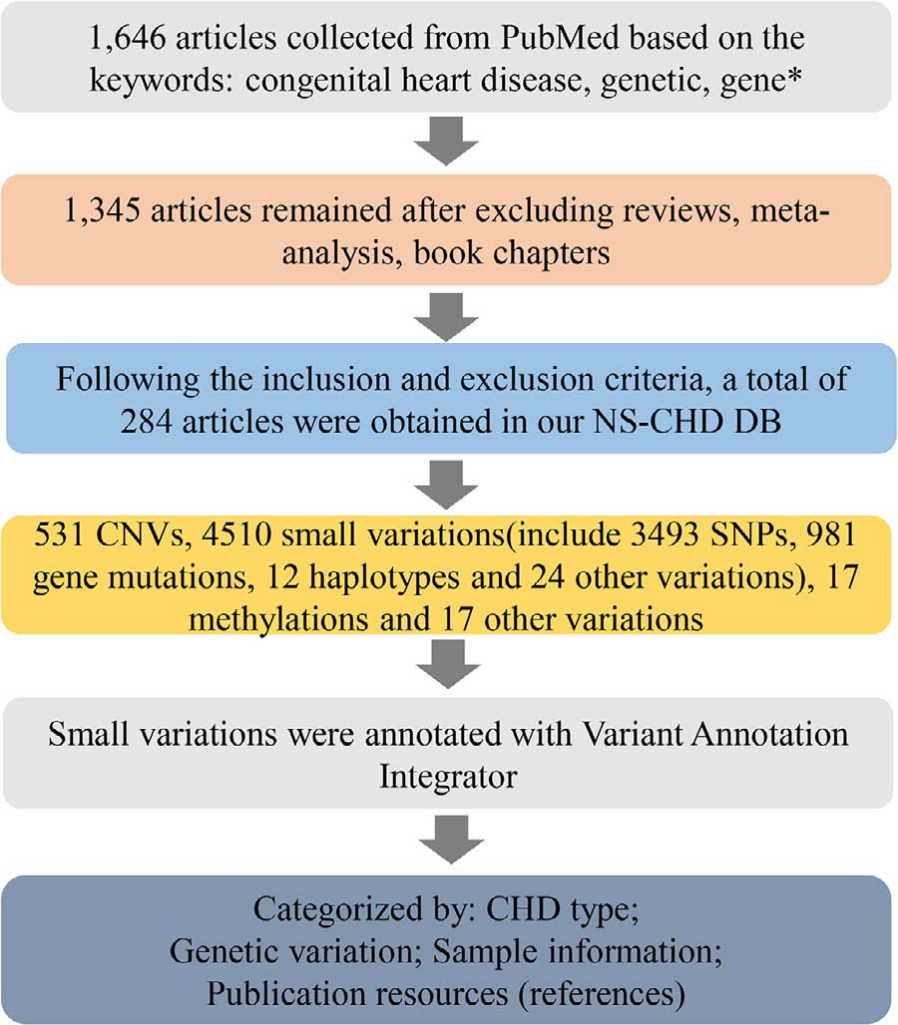
The schematic flow of the NS-CHD data collection and construction. After a series of standard selections, there were 284 articles selected from PubMed and incorporated into our database construction.

Following discussion with database construction experts, molecular biologists, clinicians, medical researchers, biostatisticians and bioinformaticians, we collected all the data for our NS-CHD genetic knowledge database (CHDGKB) from PubMed, by manual text mining. The literature searches were performed on publications prior to 31 July 2019 with the following keywords: ((congenital heart disease [Title/Abstract]) AND (genetic [Title/Abstract] OR gene^*^[Title/Abstract])). As a result, 284 out of 2054 publications from 1998 to 2019 were selected for our NS-CHD database. The data collection work flow is depicted in Figure 1.

### Inclusion and exclusion criteria

For inclusion in the CHDGKB, a study had to meet the following criteria: (i) all the patients incorporated into the CHDGKB presented with clinical features of CHD and had echocardiographic evidence or surgical records and (ii) all data in our CHDGKB was collected based on the inclusion criteria requiring that all the included studies were performed in accordance with their approved institutional guidelines and with the informed consent signed by all human study subjects. We hereby confirm ethical statements and approvals for all study data that we collected.

Genetic association data were excluded when (i) patients exhibits clinical features of CHD without echocardiographic evidence or any other examination results of heart structure; (ii) patients have any confirmed chromosomal abnormalities or syndrome-associated cardiovascular abnormalities; (iii) patients have other known complication, such as Noonan, DiGeorge, Holt-Oram, Marfan, Chat and other syndromes; and (iv) patients have established obvious environmental risk factors for CHD, such as maternal illness, drug use during the first trimester of pregnancy, parental smoking or chronic exposure to toxicants and ionizing radiation.

### Database construction

**Figure 2:**
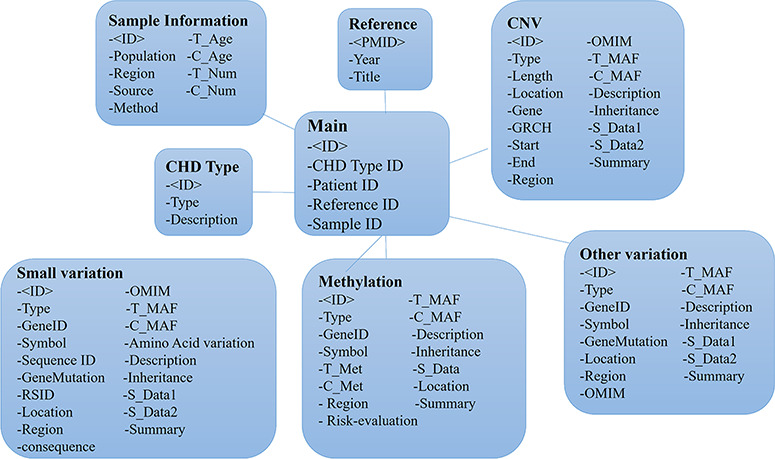
The entity relationship diagram of the CHDGKB.

The CHDGKB web interface was constructed with MySQL (5.6.19) server, Apache (2.0.61), PHP (5.2.5), HTML (5) and JavaScript. All of the web operations were implemented in the Windows operation system (64). An overview of the construction of CHDGKB is shown in Figure 2.

### Browsing and data retrieval

Users can browse the variation by choosing CHD type, variation type, gene type (protein coding or miRNA) or variation consequence (e.g. missense variant, stop gained and intron variant).

Users can search for the detailed information on genetic variants on query interfaces through the following processes:

1. Search with key gene names: users can search for the details on a given genetic variations by entering its abbreviated gene name.
2. Search with drop-down menu: users can search for any genetic variation types, CHD types/subtypes and variation consequence by selecting the terms from the drop-down menu.

### Functional enrichment analysis

Due to the complexity of subtypes of CHD, we first divided all the subtypes in our database into two main categories: isolated CHD and non-isolated CHD. Cases of isolated CHD have a single type of abnormality associated with the condition, whereas cases of non-isolated CHD have two or more types of abnormality. To further analyze the genetic factors and their correlations with isolated and non-isolated CHD, we performed Gene Ontology (GO) annotation using the database for annotation, visualization and integrated discovery (R package, ClusterProfiler). KEGG pathway was provided for an enrichment analysis and the Benjamini–Hochberg method was applied to adjust raw *P*-values. Based on the top 10 significantly enriched terms (adj. *P*-value <0.05), further studies of the associations were conducted with CHD through a literature validation.

### Protein positional conservation analyzing

The diversity of the distribution of amino acids in CHDGKB was measured by calculating entropies for sequences containing mutations at different positions in multiple sequence alignments. Mutual information was calculated to quantify positional co-variation (12).

### Data download and submission

**Figure 3:**
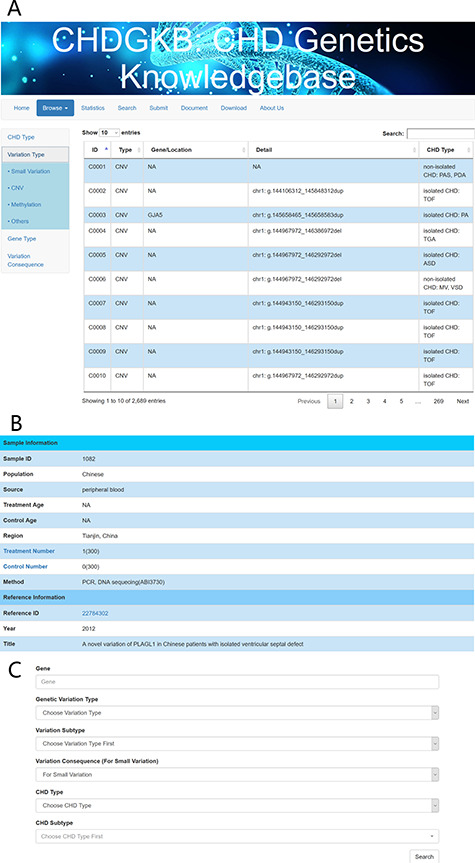
An example of the search interface with CHD subtype and precise query. Detailed information about the genetic variations can be found in the NS-CHD database. [The search interfaces are depicted in Figure **3A**, with links to the original publications (Figure **3B**). Figure **3C** show the interface for search results using key words, e.g. variations with GATA4].

All of the NS-CHD data are downloadable as Excel files (http://www.sysbio.org.cn/CHDGKB/Download.html). The search interfaces of the two search methods are depicted in Figure 3A, with links to the original publications (Figure 3B). Figure 3C shows the interface for search results using key words, with links to the original publications.

The NS-CHD research data can be submitted to community-recognized repositories at http://www.sysbio.org.cn/CHDGKB through the ‘Submit’ interface without a username or password.

## Results

### Database content and statistics

Our CHDGKB covers details from 284 individual studies conducted in more than 23 countries, with 4510 small variations, 531 copy number variations (CNVs), 17 methylations and 17 other genetic variations distributed in 370 NS-CHD subtypes. The small variations include 3493 items of SNPs, 981 items of gene mutations (NOT SNP), 12 haplotypes and 24 others. The CHDGKB contains comprehensive information on genetic variations of four groups: CHD type, genetic variation, publication resources and sample information.

**Figure 4:**
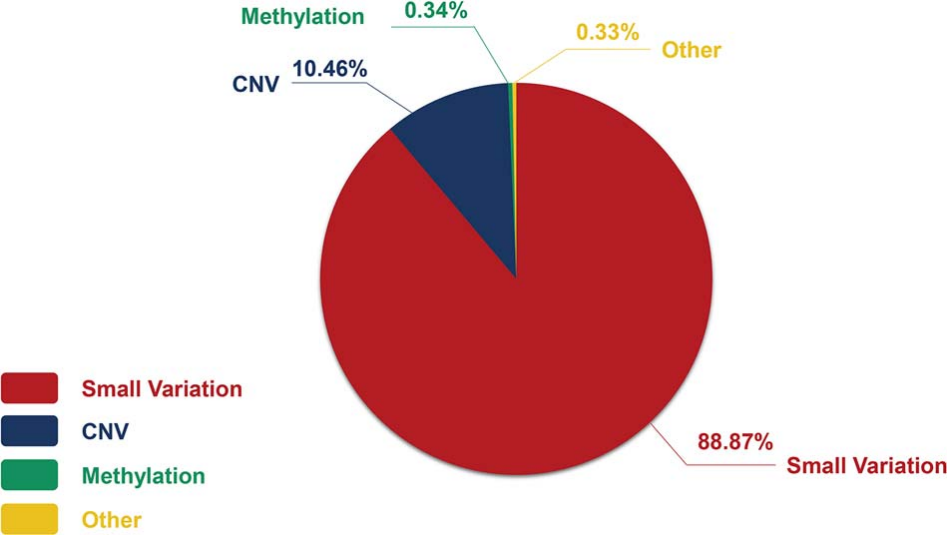
The constitution of genetic variations in the CHDGKB.

**Figure 5:**
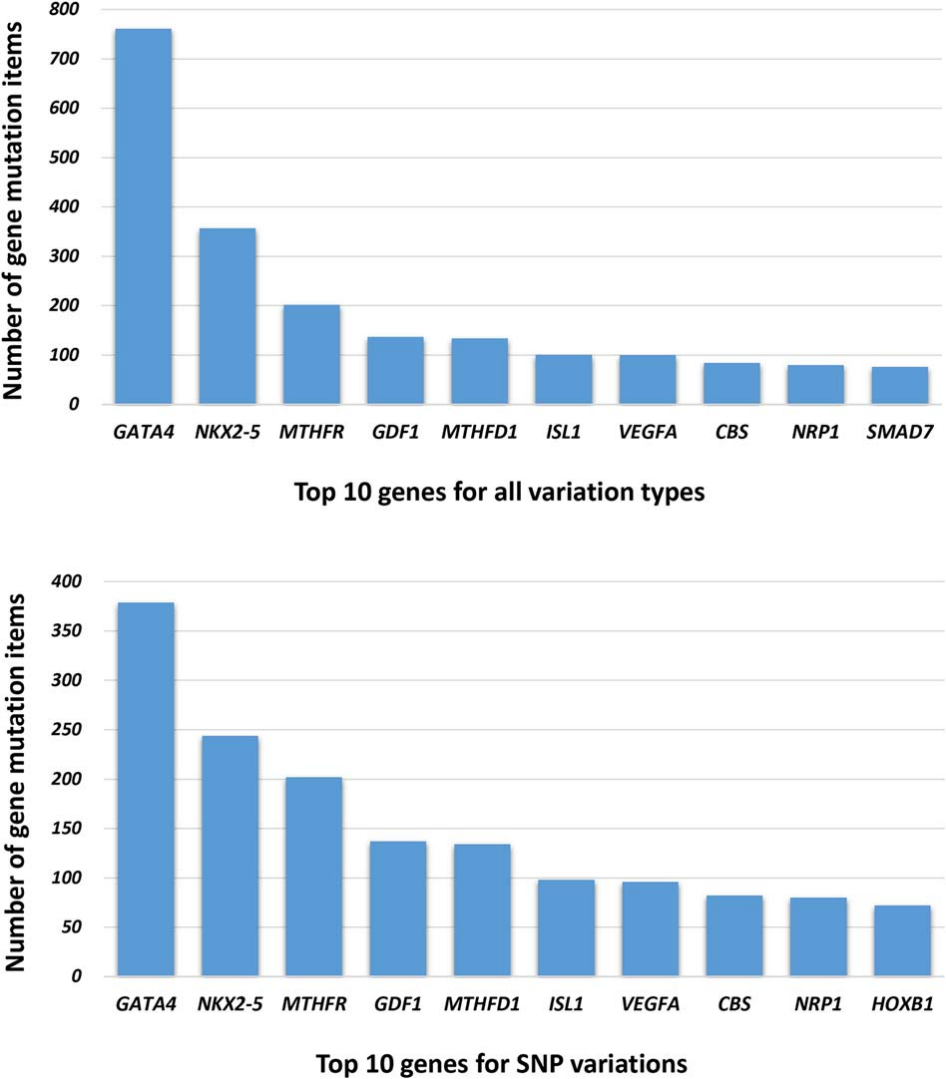
The top 10 genes associated with NS-CHD in the CHDGKB. (Figure **5A** shows the top 10 genes with all variations for NS-CHD; Figure **5B** shows the top 10 genes associated with SNP variation.)

The genetic information in our CHDGKB comprises of four variation types: CNVs, small variations, methylation and others, with majority (88.36%) of which belonging to the small variations group (Figure 4). The database contains 981 items of gene mutations, which involve 135 different genes, 3493 SNPs in 204 genes, with CNVs of 289 duplications and 237 deletions, and 34 other independent genetic variations mentioned above. The top 10 most frequently occurred genes in our database are listed in Figure 5A. Among these 10 genes, GATA4, NKX2.5, MTHFR, GDF1, MTHFD1, etc. contain not only gene mutations but also SNP variations (Figure 5B).

### Comparison with existing databases

Thus far, several public genetic databases associated with disease have been well constructed, such as ClinVar (https://www.ncbi.nlm.nih.gov/clinvar/), OMIM (https://omim.org), DisGeNet (http://www.disgenet.org/home/), Decipher (https://decipher.sanger.ac.uk), etc. As illustrated in Table 1, our database (CHDGKB) has the following advantages.

1. As a database specific for NS-CHD, CHDGKB records genetic variation information for more than 370 NS-CHD subtypes.
2. CHDGKB is a comprehensive database of variation types of gene mutation, SNP variations, copy number variations and methylation variations associated with NS-CHD.
3. The annotations in our database contain specific information for future translational applications, such as patient sample information, inheritance information and risk level for risk evaluation, diagnosis, prognosis,etc.

**Table 1:** Comparison with other databases

Database/items	CHD subtypes	Variation Types					Methylations			Locations	Inheritance	Sample information
		Gene mutation	SNP	Allele	CNV	Aneuploidy	Location	Methylation levels	Risk levels			
ClinVar	√	√	√	√	√	√	×	×	×	√	×	√
OMIM	√	√	√	√	√	√	√	√	×	√	√	√
DisGeNet	√	√	√	√	√	√	×	×	×	√	×	×
ISCA	√	√	√	√	√	√	√	√	×	√	√	×
DECIPHER v9.31	√	√	√	√	√	√	√	√	×	√	×	√
CHD GKB	√	√	√	√	√	√	√	√	√	√	√	√

Abbreviations: CHD, congenital heart disease; GKB, genetic knowledge database; InDel, insertion and deletion; SNP, single nucleotide polymorphism; CNV, copy number variation

### GO analysis and pathway mapping

**Figure 6:**
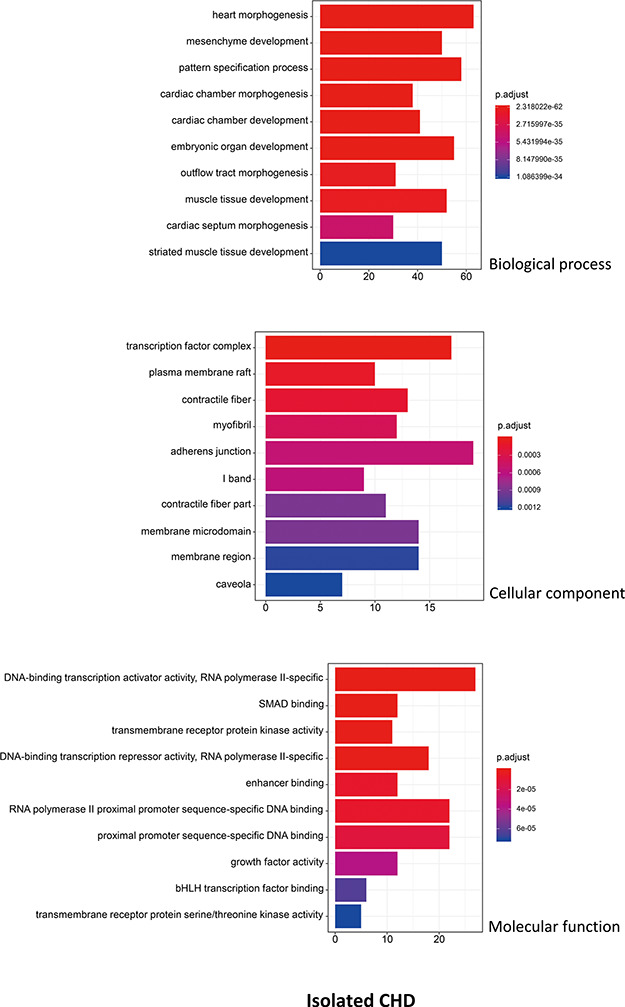
The top 10 significant enriched GO terms for isolated CHD. The statistical significance level (p.adjust, adjusted *P*-value) was depicted as different color.(*X*-axis indicated number of enriched genes; *Y*-axis indicated GO terms.)

**Figure 7:**
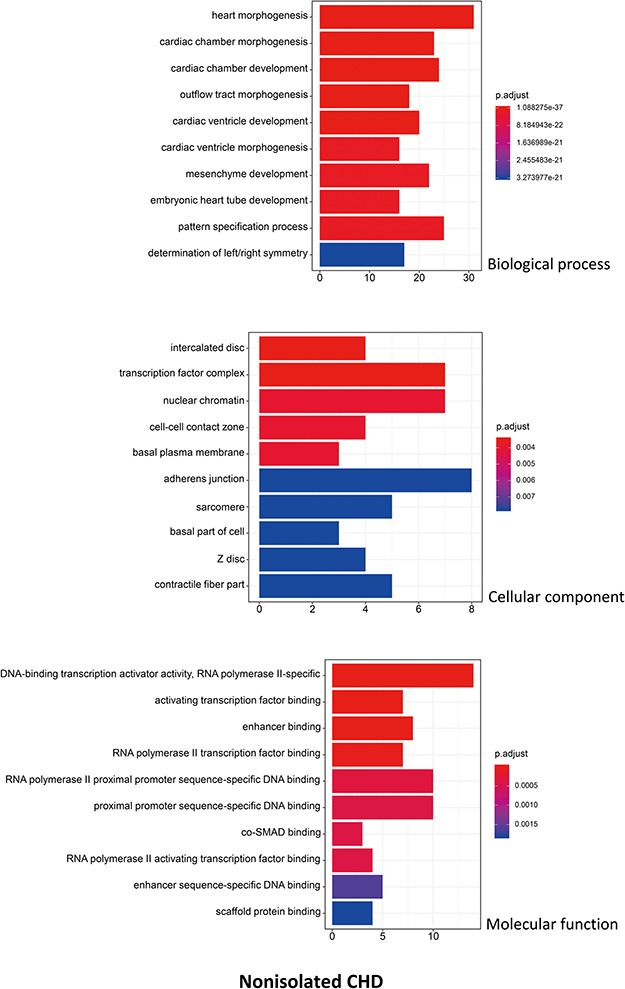
The top 10 significant enriched GO terms for non-isolated CHD. The statistical significance level (p.adjust, adjusted *P*-value) was depicted as different color. (*X*-axis indicated number of enriched genes; *Y*-axis indicated GO terms.)

**Figure 8:**
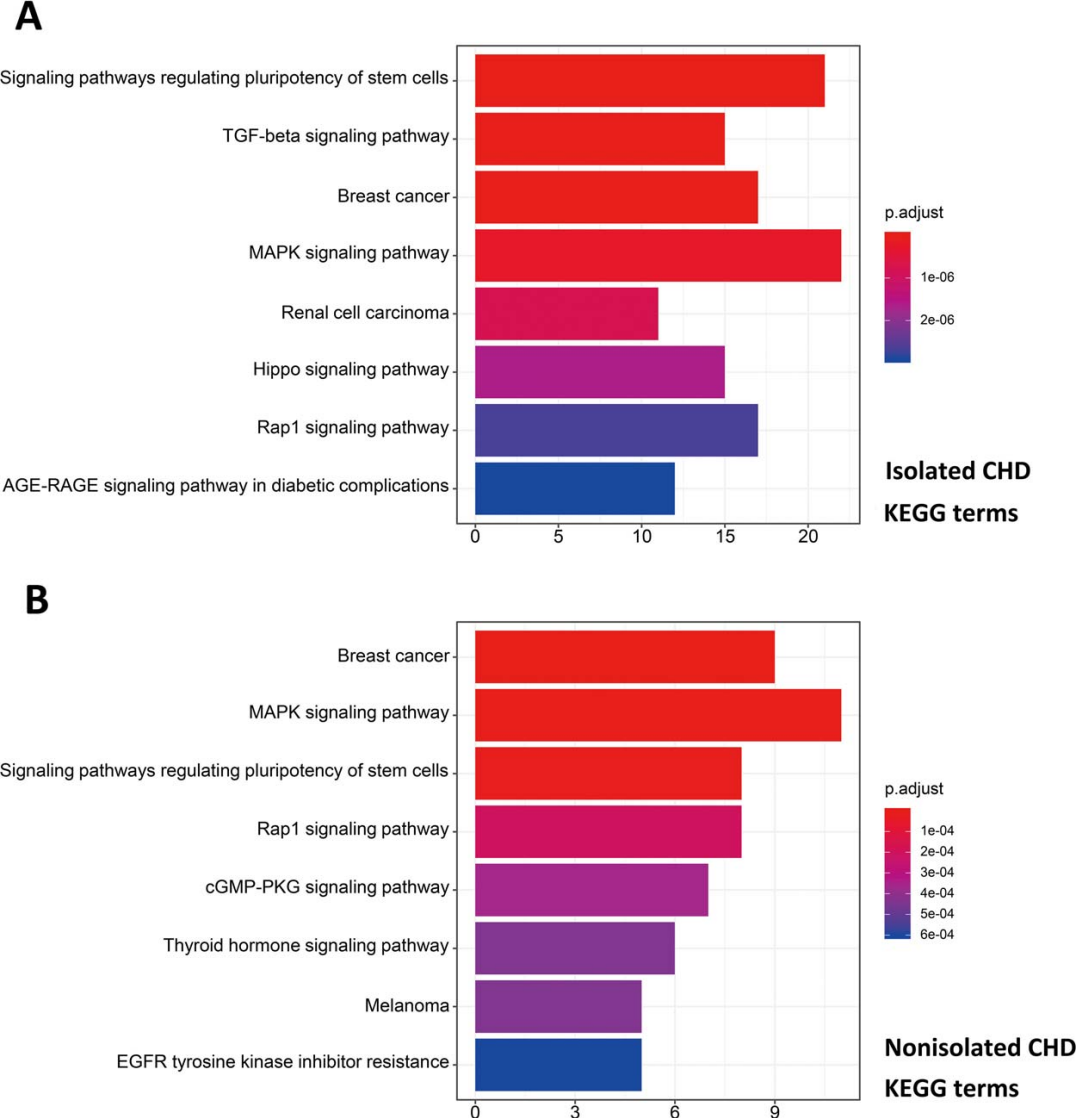
Pathway enrichment analysis for genetic variations of NS-CHD. The statistical significance level (p.adjust, adjusted *P*-value) was depicted as different color. (Figure **8A**, the top eight significant KEGG terms for isolated CHD; Figure **8B**, the top eight significant KEGG terms for non-isolated CHD. *X*-axis indicated number of enriched genes; *Y*-axis indicated enriched pathways.)

R package ClusterProfiler was used to the GO analysis of isolated and non-isolated CHD at three levels: biological process (BP), cellular component (CC) and molecular function (MF). The top 10 significant enriched terms (*P* < 0.05) of each level and number of genes for the two main kinds of CHD are summarized in Figures 6 and 7, respectively. At the BP level, for both isolated and non-isolated CHD, the most significant terms were mainly related to cardiac chamber morphogenesis and development, mesenchyme development and heart morphogenesis. Researchers have demonstrated that mutations in some key genes, such as TBX20 (13), NKX2.5 (14) and CELSR1 (15), play a vital role in the heart morphogenesis process through down-regulation or up-regulation of the correlated genes. Furthermore, Bose *et al.* (16) revealed that mutations in non-coding regions of GATA4 could also affect the process during fetal heart development. At the CC level, the enriched terms for the two kinds of CHD were focused on adherents junction (17), contractile fiber part (18) and transcription factor complex (16). It has been shown that mutations occur in the whole coding region and splice junction sites of the PITX2c gene, which encodes paired-like home domain transcription factor 2 and is crucial for normal cardiovascular morphogenesis. Also, it is well known that GATA6, which is also a gene encoding a zinc finger transcription factor, plays a significant role in the core cardiac transcriptional factor pathway. At the MF level, results of significant enriched terms for isolated and non-isolated CHD are mapped with not only DNA-binding transcription factor activity (19), enhancer binding (20) and SMAD binding (21) but also proximal promoter sequence-specific DNA binding (22). Meanwhile, we provided KEGG pathways for the enrichment analysis. The top eight significant enriched terms of KEGG pathways along with number of genes for isolated and non-isolated CHD can be seen in Figure 8. Series of MAPK signaling pathways (23), signaling pathways regulating pluripotency of stem cells (24) and Rap1 signaling pathway (25) are essential for occurrence of both isolated and non-isolated CHD.

### Protein positional conservation analyzing with 3-fold conservation algorithm

To explore the pathogenesis of NS-CHD at the molecular level, we applied this three-type conservation algorithm into analyzing some important proteins, e.g. transcription factor GATA-4, on which 32 disease-causing mutations were reported over 100 times and well collected in our database. We got MSA for this protein from protein family (https://pfam.xfam.org/) (26). It contains 313 alignments and the sequence length is 205. Three kinds of conservation values are calculated and listed in Table 2–4.

**Table 2:** Type I and II residue conservations on protein transcription factor GATA-4^*^

No	Type I conservation: 20 amino acid alphabets			Type II conservation: divided amino acids with physicochemical properties (six amino acid categories)		
	Residue	Rank	Information value	Residue	Rank	Information value
1	43R	6	2.936	154F	5	2.955
2	6A	24	2.666	110F	11	2.920
3	44V	25	2.659	95D	20	2.575
4	51L	27	2.647	193P	29	1.992
5	5L	30	2.597	116S	37	1.614
6	193P	41	2.326	118A	45	1.390
7	55Q	45	2.280	197N	49	1.356
8	110F	47	2.211	150G	51	1.300
9	46S	57	2.088	90S	66	1.093
10	116S	59	2.073	96G	75	1.006
11	95D	60	2.073	163P	76	0.995
12	33A	63	2.015	93G	83	0.833
13	118A	70	1.897	125A	86	0.781
14	9A	94	1.741	144A	87	0.777
15	163P	98	1.720	167A	90	0.679

^*^The table only list reported disease-causing mutation residues with information value ranking top 100.

**Table 3:** Type III mutual residue conservations on protein transcription factor GATA-4^*^

No	20 aas alphabet			Physicochemical properties (six categories)		
	Residue1	Residue2	Mutual information value	Residue1	Residue2	Mutual information value
1	118A	119A	182.031	163P	164A	184.663
2	117L	144A	139.724	118A	120A	176.495
3	117L	154F	163.434	90S	167A	120.454
4	163P	164A	179.671	118A	139A	121.935
5	167A	180S	138.668	163P	167A	119.326
6	93G	106P	122.115	157S	163P	115.311
7	154F	164A	131.230	167A	180S	126.966
8	154F	163P	124.885			
9	117L	121A	134.008			

^*^The table only list pairs with top 20 high mutual information containing reported disease-causing mutation residues.

**Table 4:** High conserved triplet with disease-causing mutations on protein Transcription factor GATA-4^*^

No	20 amino acid alphabets				Physicochemical properties (6 amino acid categories)			
	Residue1	Residue2	Residue3	Triplet rank	Residue1	Residue2	Residue3	Triplet rank
1	97A	117L	154F	2	98A	102P	163P	1
2	97A	123A	154F	3	102P	109S	163P	3
3	98A	102P	163P	4	102P	159S	163P	5
4	99Y	105S	154F	7				
5	99Y	107R	117L	8				
6	99Y	117L	154F	11				
7	99Y	136G	154F	12				
8	105S	107R	154F	17				
9	105S	117L	154F	19				
10	105S	123A	154F	20				

^*^The table only list the triplets among top 20 most conserved one.

## Discussion

Prior to our current study, several systematic studies had been focused on psychiatric disorders, such as the bipolar affective disorder, the attention deficit disease and autism (27–30). Researchers have also developed genetic databases on Parkinson’s disease, diabetic retinopathy and hepatocellular cancer (31–33). Furthermore, there were similar diseases-associated gene/genetic variation database for hypertension, obesity (34), diabetes (35), coronary artery disease (36) and aortic aneurysm (37). Here we present, to our knowledge, the first open database for NS-CHD genetic variants, with numerous predictive functions. This database represents an up-to-date, comprehensive synopsis for the NS-CHD genetics research. It can be used for the SNP analysis, as well as meta-analysis such as ethnicity-specific meta-analysis, on the NS-CHD risk-gene candidates based on all 981 items of gene mutations and 3493 items of SNP variation, following stratification for different countries.

Using the GO annotation, we performed further bioinformatic analyses on the isolated and non-isolated CHD. At the BP level, for the enriched terms ‘heart morphogenesis and cardiac chamber development’ shared by both two CHD types, the BP of cardiac septum morphogenesis was mainly related to isolated CHD. For example, NKX2.5 influences the process of heart growth by up-regulating its target genes, including those involved in the atrial septal defect (14) and those necessary to maintain chamber-specific identity in both the first and second heart field (38). On the other hand, studies on non-isolated CHD mostly focused on regulation of pathways via vital genes. Mutation in CELSR1 (P870L) was shown to correlate with various CHD subtypes, including septation or conotruncal defects (15) through up-regulation of the PCP pathway and the canonical WNT signaling in cells. This further demonstrates the complex functions of genes and their interactions involved in the cardiac ventricular development (39) during the BP of CHD. At the CC level, isolated CHD was focused on the membrane region, which was probably due to that heart membrane microdomains are enriched in chaperones, cytoskeletal-associated proteins, enzymes and protein involved in signal transduction pathway (40). However, it has been demonstrated that the main morphologic of cardiocyte differences were dependent on nuclear chromatin activity/stainability and nuclear breadth (41), which can explain why the non-isolated CHD was correlated with the CC terms of nuclear chromatin. At the MF level, except for the shared significant terms, the enriched terms in isolated CHD are mainly correlated with transmembrane receptor protein kinase activity (40) and growth factor activity, which both play an important role in the process of message transfer to regulate and affect transcription (42, 43). The phenotypes of non-isolated CHD are more complex than those of isolated CHD in terms of nucleotide binding and interaction (44). Consequently, the distinct enriched terms of non-isolated CHD are activating transcription factor binding and protein binding.

On the basis of the associated pathways in isolated CHD, studies of immunohistochemistry for molecules in the TGF-β signaling pathway have demonstrated that ongoing tissue remodeling of the coronary artery disease after the acute injury and confirmed the importance of the TGF-β signaling pathway in this process (45). Besides, there is another pathway involved in the process of isolated CHD, the Hippo signaling pathway, which can regulate embryonic cardiomyocyte proliferation and heart size during development through YAP (46). Studies have revealed its mechanism was that Hippo crosstalk with Wnt/β-catenin signaling can play a critical role in mediating the positive effect of YAP on cell cycle-related gene expression and cardiac overgrowth (46, 47). Compared with the influence in the initial process of heart development via hippo signaling pathway, the different significant enriched pathway correlated with non-isolated CHD focused on the cGMP-PKG signaling pathway, which are both recognized modulators of cardiac function and the chronic stress response (48). Tsai *et al.* (49) have revealed that when their enhancing relax, cGMP/PKG serve as a myocardial brake, countering cAMP stimulation and independently signaling alternative pathways to blunt contraction and growth. Furthermore, studies have suggested the various cGMP regulating phosphodiesterases and confirmed their proposed interactions with cGMP, cAMP and PKG myocyte target (49, 50). The complex interactions among these pathways subsequently involved in the occurrence of non-isolated CHD via reducing maladaptive hypertrophy, improving cell survival, regulating signaling and mitochondrial function, protecting against ischemia/reperfusion injury and blunting the stimulatory effects of catecholamines (48, 51).

Based on our previous study (12), we made further analysis of function mechanism via 3-fold conservation algorithm toward proteins at molecular level. Entropy calculations are used to identify types I and II conservation. For type I conservation, the normal alphabet of 20 amino acids is used while the amino acid alphabet is divided into six physiochemically in type II conservation (52). The six categories are hydrophobic (V I L F M W Y C), negatively charged (D E), positively charged (R K H), conformational (G P), polar (N Q S) and (A T).

For the types I conservation or single residue conservation (Table 1), there are 15 residues with reported mutations are regarded as highly conserved (top 10). The residue 43R (ranking 6th of all 205 residues) is seven times reported as disease related in our database; the mutation in this residue is R43W. If we divided amino acids with physicochemical properties (type II conservation), also 15 residues with disease-causing mutations are regarded as highly conserved (top 100), six of which are also regarded as highly conserved in type I (110F, 95D, 193P, 116S, 118A and 163P). The residue 154F is found to be most highly conserved, ranking fifth of all positions, deletion mutation in this residue is three times reported. For the type II conservation or the mutual conservation, we calculated information values focused on specific pairs of residues. The disease-causing mutations related to type II conservation are listed in Table 2. There are nine such pairs of residues for the normal alphabet of 20 amino acids and eight pairs for the six physicochemical alphabet which have top 20 mutual information. These pairs contain nine different disease-causing mutations reported in the database. The pair 118A and 119A has third biggest mutual information value among all pairs, and the disease-related mutation between the two residues is an insertion. For physicochemical properties, the pair 163P and 164A has the second highest mutual values, and there was a substitution P163S reported. Furthermore, we can find conserved triplets, which means co-variation among three residues, according to the mutual information. Those highly conserved triplets with six disease-causing mutations are listed in Table 3. The two residues, 154F and 163P, play most important roles in triplet conservation in 20 amino acids alphabet and six physicochemical properties group, separately.

We made an analysis of GATA4 variation as an example, which was reported as one of the most genetic variation associated with NS-CHD. We found that some single residues with high conservation value might not be important in co-variation or triplet, which means co-variation with three different residues. Some residues might need further analyzation in future, like 117L, 154F and 118A, because these residues are included in several pairs of co-variation and triplets.

In brief, our CHDGKB provides comprehensive results, with which we can perform statistical and systematic analyses to further our understanding of how genetic factors influence the pathogenesis of CHD. For example, GATA factors regulate a large number of cardiac genes, including NPPA, NPPB, MEF2c, NKX2.5, BMP4, MYH6 and MYH2 (53), and the complex gene interactions, regulations and the resulting functional variation of proteins all affect the progression of CHD. Our future direction will be to explore the different enriched gene network and study the mechanisms involved in the various subtypes of NS-CHD. Moreover, our CHDGKB also provides homogeneous results using a statistical analysis to enable investigations into the correlations between genotypes and phenotypes of NS-CHD. For example, researchers can examine mutations in GATA4 and NKX2.5 and their correlations to specific NS-CHD subtypes. Through browsers such as the Human Genetic Variation Browser (54), we can study the allele frequency of genetic variations that cause non-synonymous amino acid changes in patients included in our database. Using statistical methods such as the weighted genetic score, we can calculate the genetic score (55) and logistic regression model (56) on the information pulled from our CHDGKB. This will greatly improve risk assessment and prediction of NS-CHD in the near future.

There are some limitations in our NS-CHD database, and future improvements have already been planned. The current version documents 5345 variations in populations from 24 countries until July 2019, and it will be continually updated. Most of the data in the current version came from the Asian population, which was the actual distribution in PubMed. Furthermore, the genetic information in our database will be expanded to include gene expression, functional variations and other more comprehensive genetic parameters in future, in order to further investigate the relationships between NS-CHD and genetic variations. Using our database, additional systematic analyses on the molecular mechanisms of NS-CHD are also planned to improve and expand its clinical applications.

## Author Contributions

The authors’ responsibilities were as follows: Bairong Shen, Lan Yang and Xingyun Liu designed the research; Lan Yang, Xingyun Liu, Yalan Chen and Yuxin Lin performed literature search, selection and data extraction; Xingyun Liu constructed the database; Yang Yang and Yongquan Chen performed the functional analysis of the amino acid mutations. Lan Yang, Yan Sun and Bairong Shen write the manuscript; Bairong Shen conceived and supervised the work. All the authors completely consented with all the data in the study, critically revised the manuscript for important intellectual content and approved the final version.

## Additional Information

**Data resource access:** http://www.sysbio.org.cn/CHDGKB/

## Conflict of interests

None declared.
